# Litigation Following Surgery for Acute Traumatic Facial Injuries

**DOI:** 10.7759/cureus.72660

**Published:** 2024-10-29

**Authors:** Mattie R Rosi-Schumacher, James D O'Donnell, Adrian A Ong, David A Sherris

**Affiliations:** 1 Otolaryngology - Head and Neck Surgery, University at Buffalo Jacobs School of Medicine and Biomedical Sciences, Buffalo, USA; 2 Otolaryngology, Brookdale University Hospital Medical Center, New York, USA

**Keywords:** facial fracture, facial injury, facial plastic surgery, facial trauma, facial trauma surgery, lawsuit, litigation, malpractice, maxillofacial trauma, plastic surgery

## Abstract

Introduction: The knowledge of malpractice claims after surgery for acute traumatic facial injuries can inform surgeons about potential litigation risks, case complications, and reasons for patient dissatisfaction.

Methods: The Westlaw Campus Research and LexisNexis legal databases were searched for all available court decisions associated with cases brought to suit following surgery for traumatic facial injuries. The plaintiff characteristics, date, location, specialty of the defendant, allegations, and adjudicated case outcomes were obtained from case documents and analyzed.

Results: There were 27 relevant judicial cases included between 1985 and 2023. The cases were tried in 16 different states, with New York having the greatest number of cases (25.9%). About half of the plaintiffs were male (51.9%), but the age was unknown in 55.6% of cases, while 29.6% were >18 years old and 14.8% were pediatric patients. The most common method of injury was a motor vehicle accident (44.4%), followed by assault (14.8%). Otolaryngologists were cited in 22.2% of cases, while 33.3% of cases involved a plastic surgeon. The most common reasons for negligence were malocclusion resulting from a fracture (nine cases, 33.3%) and poor post-operative management (six cases, 22.2%). Twelve (44.4%) adjudicated cases were decided in favor of the surgeon.

Conclusion: The most common reasons for litigation following acute traumatic facial injuries were malocclusion and issues during post-operative management, such as surgical site infections. The majority of cases identified and analyzed in this study were adjudicated in favor of the patient.

## Introduction

The treatment of facial trauma injuries has important implications for both functional and aesthetic outcomes and is typically entrusted to sub-specialized physicians with training in maxillofacial trauma surgery. As in any other field, when patients perceive poor outcomes or sub-standard care, physicians may be subject to malpractice litigation, which has wide-ranging negative consequences.

In the five states with the highest malpractice risk, 68% of physicians reported engaging in defensive medicine [1]. It is estimated that 2.9% of costs on in-patient medical services are purely defensive, and another 10.1% are somewhat defensive [2]. Across specialties, greater average spending by physicians was associated with a reduced risk of incurring a malpractice claim [3]. For example, among internists, the probability of experiencing an alleged malpractice incident in the following year ranged from 1.2% to 1.7% in the bottom spending fifth ($19,725 per hospital admission) to 0.2% to 0.5% in the top spending fifth ($39,379 per hospital admission) [3]. In six of the specialties, a greater use of resources was associated with statistically significantly lower subsequent rates of alleged malpractice incidents [3].

The contributing factors to medical malpractice claims differ substantially based on medical subspecialty and type of procedure. According to Jena et al., plastic surgery is in the top five medical specialties that are most likely to be sued among other surgical specialties [4]. Additionally, one study found that those plastic surgeons who identified their expertise as “reconstructive” were five times more likely to be sued for malpractice than their counterparts within the same field [5]. Considering these facts, those surgeons who treat traumatic facial injuries in their practice may be at a unique risk of litigation.

The rate of malpractice claims paid on behalf of physicians in the United States (US) has declined substantially since a peak of 16,116 payments in 2001 down to 6,979 payments in 2022 [6]. The passage of traditional tort reforms such as damage caps and statutes of limitation is a possible explanation for why the rate of claims paid on behalf of physicians is declining [7]. Another factor may be due to the under-reporting of claims by corporations and the growing prevalence of resolution through direct patient compensation without a written claim [7]. Even with this decrease in claims, there is an impressive $2.9 billion in payments made to patients or their families [6]. This amount only represents a portion of the toll these lawsuits exact on physicians, families, and the public in general.

The evidence regarding the impact of malpractice litigation on surgeons in the field of facial trauma is currently lacking. The objective of this study was to characterize medical malpractice suits after surgery for facial trauma and to describe associations between patient and provider demographics, geographic region, legal details, and case outcomes.

## Materials and methods

**Figure 1:**
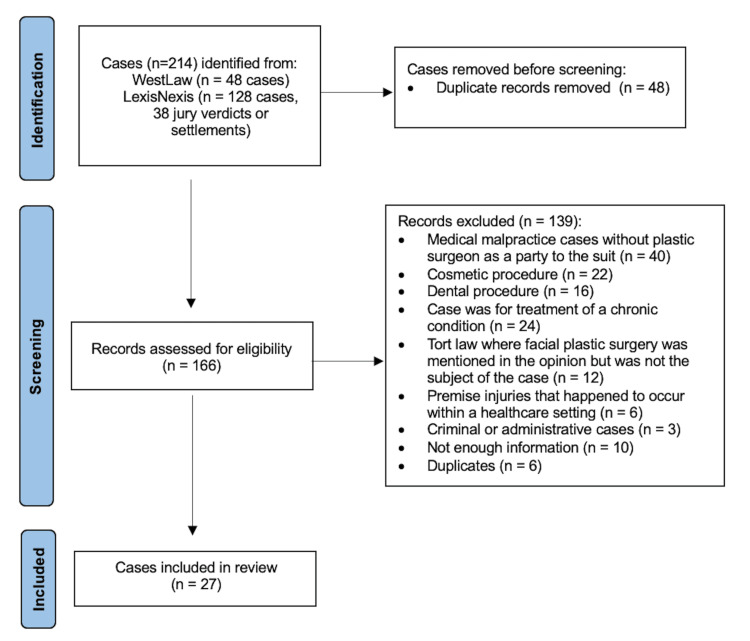
Database search and criteria for excluded cases.

This study was a retrospective review of online legal databases including Westlaw Campus Research (Thompson Reuters, New York, NY) and LexisNexis (RELX Group, New York, NY). The study used publicly available data and therefore was deemed exempt from approval by the Institutional Review Board. The Westlaw database contains jury verdict reports and summary statements for cases that were adjudicated in secondary courts. LexisNexis includes case settlements from higher court cases as well as lower court verdicts and settlements. The databases were searched in February 2023 for all state and federal legal cases pertaining to facial trauma surgery in the United States. The following advanced search criteria were used: “(‘medical malpractice’ ((fracture OR laceration OR broke) [within the same sentence as] (face OR facial OR zygoma OR zygomatic OR zygomaticofacial OR nasal OR cheek OR jaw OR mandible OR ethmoid OR zygomaticomaxillary OR maxilla OR maxillary OR panfacial OR LeFort)). Cases were included if they involved surgical repair of an acute traumatic facial injury and if the physician was a party to the case. Exclusion criteria included the following: cosmetic procedures, dental procedures, treatment for a chronic condition, premise injuries in a healthcare setting, and criminal or administrative cases. Inclusion and exclusion criteria were applied to all cases that were assessed for eligibility (Figure 1).

Data was extracted from each case including plaintiff demographics and characteristics, location of the case, specialty of the defendant, type of injury and surgery involved, and stated reasons for alleged malpractice. Jury verdicts were noted along with financial awards. Appeals data were collected when available. Descriptive statistics were utilized to describe the data as percentages of total cases.

## Results

A total of 214 cases were identified in the WestLaw and LexisNexis database searches. After excluding duplicates and cases that did not meet the inclusion criteria, 27 cases dated from 1985 to 2023 were included. The plaintiffs' characteristics are presented in Table 1. The plaintiffs' ages ranged from two to 67 years old, and 51.9% of them were male. The defendants’ specialties included general surgery (9, 33.3%), plastic surgery (9, 33.3%), otolaryngology (6, 22.2%), oral maxillofacial surgery (1, 3.7%), orthopedic surgery (1, 3.7%), and emergency medicine (1, 3.7%). 

**Table 1:** Characteristics of the plaintiff and injuries.

Plaintiff Characteristics	N (%) Total N=27
Gender	
Female	11 (40.7)
Male	14 (51.9)
Unknown	2 (7.4)
Age	
Unknown	15 (55.6)
0-18	4 (14.8)
19-35	4 (14.8)
36-65	3 (11.1)
65 and older	1 (3.7)
Method of Injury	
Motor vehicle accident	12 (44.4)
Assault	4 (14.8)
Fall	3 (11.1)
Dog bite	3 (11.1)
Bicycle	2 (7.4)
Horseback riding accident	1 (3.7%)
Unknown	2 (7.4)
Number of Significant Injuries in Suit	
Single	22 (81.5)
Multiple	5 (18.5)
Type of Injury	
Mandible fracture	13 (41.9)
Orbital fracture	4 (12.9)
Nasal bone fracture	4 (12.9)
Facial laceration	4 (12.9)
Zygoma fracture	3 (9.7)
Maxilla fracture	2 (6.5)
LeFort fracture	1 (3.2)

In five cases, the suits were brought by individuals other than the person suffering the injury including the following: two cases in which a patient died due to their injuries and the suit was brought by their estate, one case of a child who died due to their injuries and the suit was brought by their parents, and two cases initiated by the parents of underage children who could not bring the case to court on their own. 

The most common mechanism of injury was sustained from a motor vehicle accident. There were a total of 31 traumatic injuries in the cases. The most common was a mandible fracture, which represented 13 (48.1%) cases. Many of the cases did not cite a specific surgical procedure performed; however, of the cases that did, six (22.2%) involved open reductions of the mandible, five (18.5%) involved complex facial sutures, four (14.8%) involved functional rhinoplasties, and three (11.1%) cases involved skin grafting. In three of the cases, there was no surgery performed when one was indicated.

**Figure 2:**
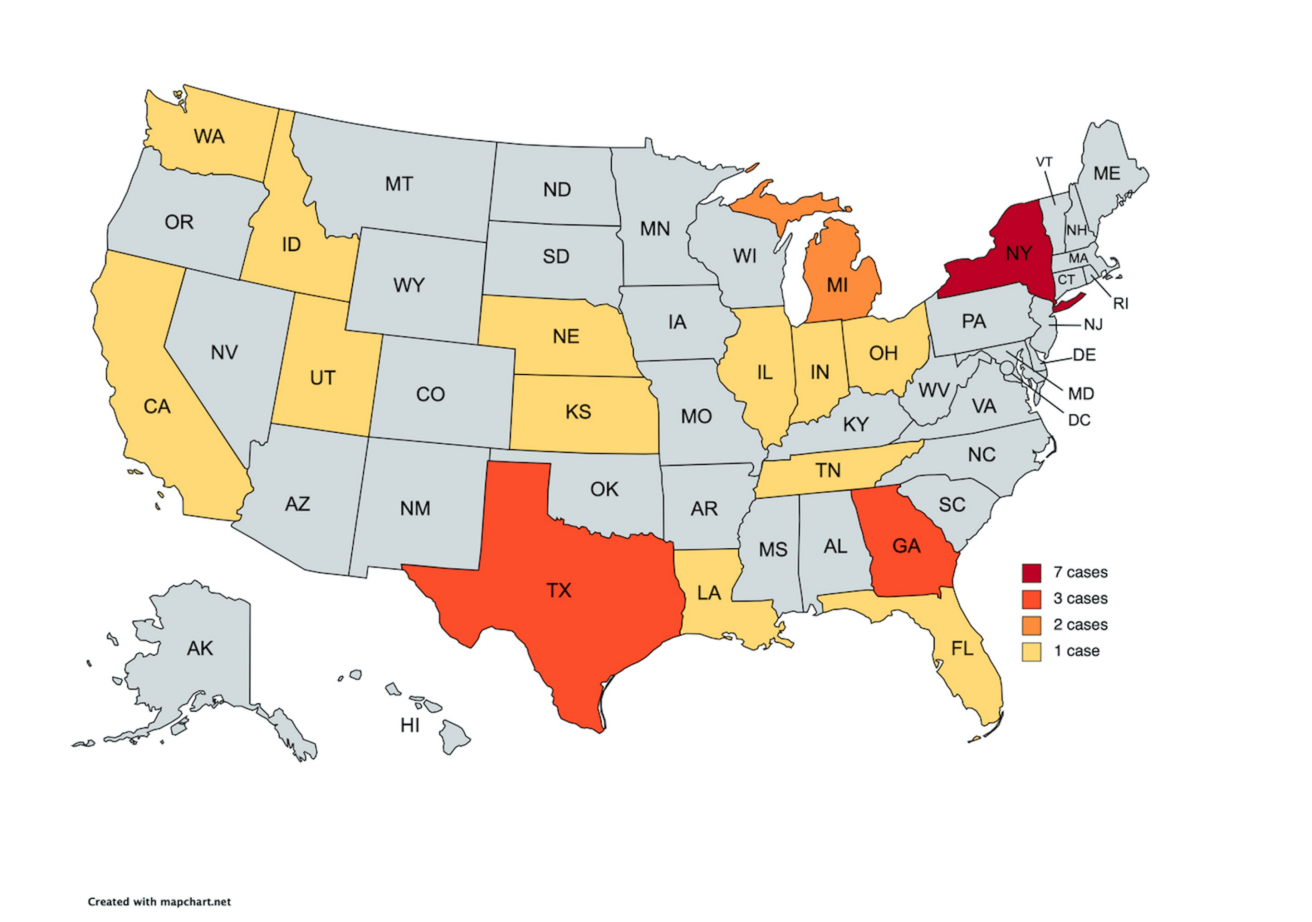
Number of cases tried per state.

The cases were tried in 16 different states, with New York having the greatest number of cases (7, 25.9%) (Figure 2). Georgia and Texas each had three (11.1%) cases while Michigan had two (7.4%) cases. The remaining twelve states all had a single case each. Physicians had a favorable verdict 66.7% of the time in the West, 60% of the time in the Southeast, 50% of the time in both the North and Southwest, and 0% of the time in the Midwest and Mountain Plain states. 

**Figure 3:**
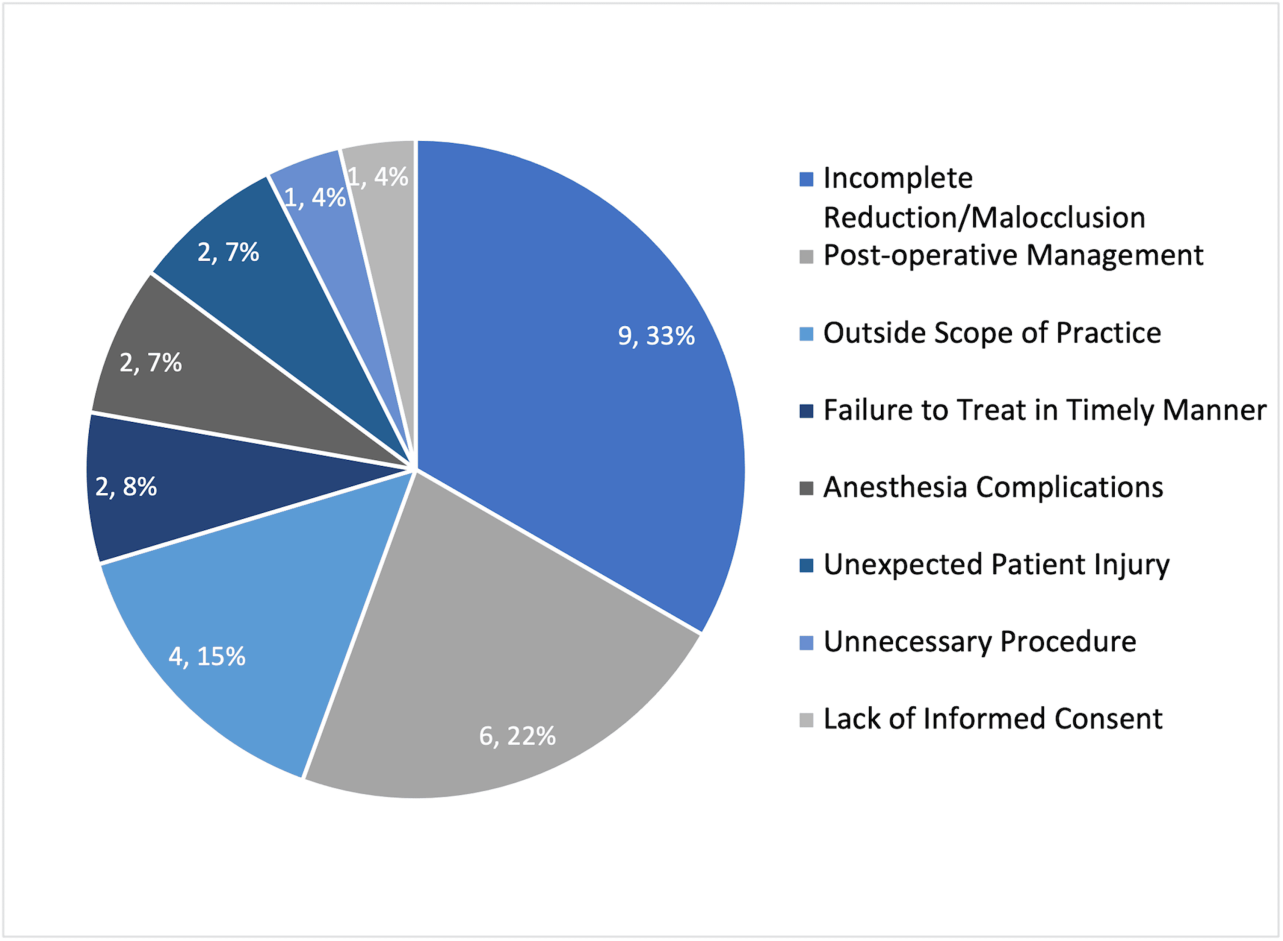
Reason for negligence cited in the malpractice claim (case number, %).

The issues of negligence, whether founded or not, that led to litigation are detailed in Figure 3. All but one of the claims of incomplete reduction/malocclusion were regarding mandible fractures, whereas the last was after the reduction of both nasal and maxillary fractures. Post-operative complications represented the second most common reason for litigation. There were four cases of infection resulting from the following: retained nasal packing (two cases), infected teeth in a mandibular fracture (one case), and post-operative infection after mandible fracture in a patient who left the hospital against medical advice (one case). One of the post-operative management claims was brought for intractable post-operative pain after nasal surgery, for which the patient wanted a second operation that was deemed not indicated by the surgeon. 

Of all the cases included in this report, three resulted in death. One patient's death was a result of a devastating injury not related to the facial fractures (subdural hemorrhage). In the other two cases, the death was deemed avoidable and directly caused by errors in medical treatment. In one case a patient suffered from post-procedure propofol infusion syndrome that had been ordered by the plastic surgeon for post-operative sedation for a prolonged period of time. In the last case, the physician failed to examine a patient who became hypoxic after surgery and subsequently died of acute respiratory failure.

Nine cases (33.3%) reported an associated monetary reward ranging from $19,960 to $1,900,000. All the cases that resulted in awards over $1,000,000 involved physician inattentiveness including failure to perform follow-up examination, failure/delay of appropriate diagnosis, failure to recognize other co-morbid injuries resulting in accidental death, and failure to seek consultation from other specialties for additional injuries. 

**Figure 4:**
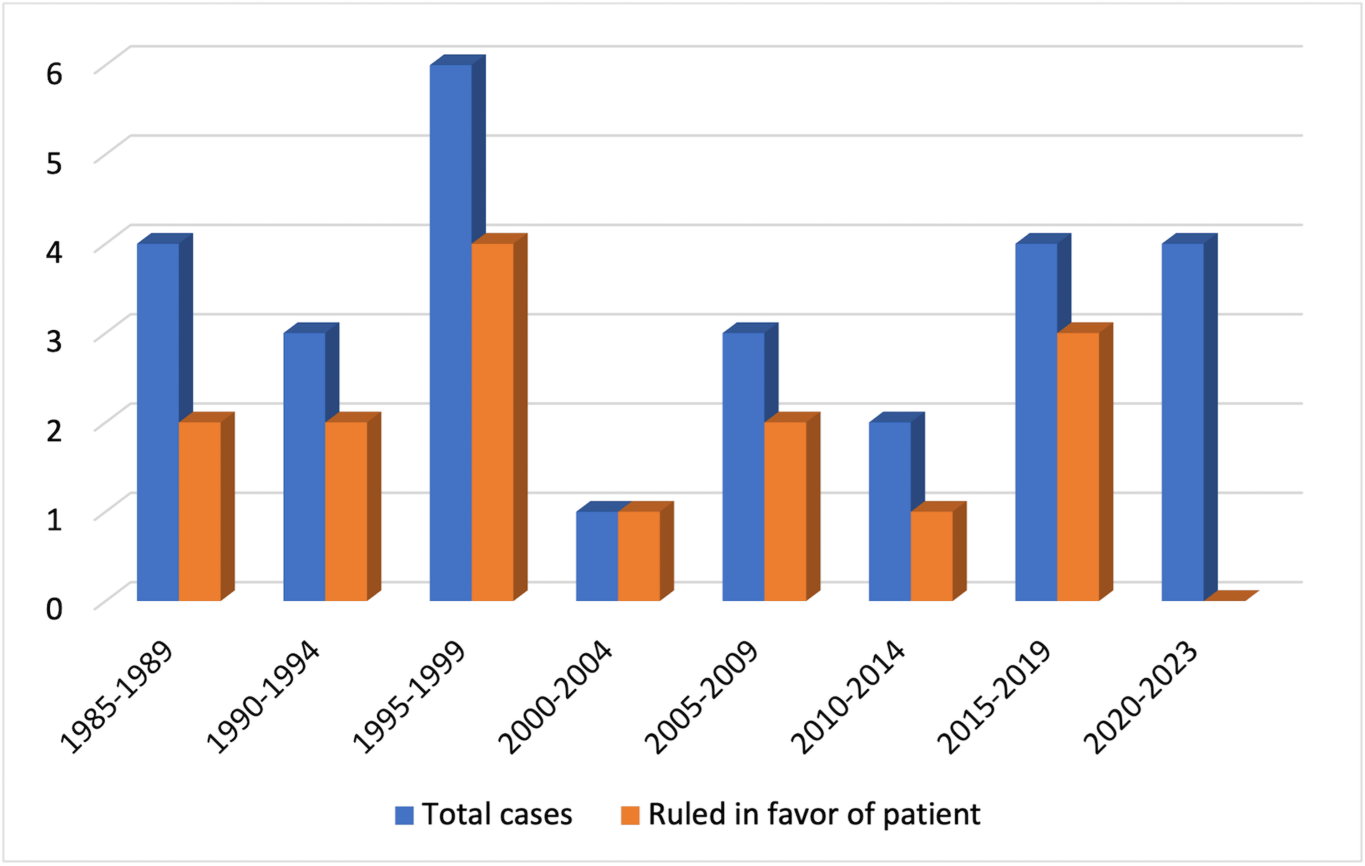
Total number of cases by year and number of cases ruled in favor of the patient.

The cases examined in this study took place from 1985 to 2023 (Figure 4). In all, 12 (44.4%) adjudicated cases were decided in favor of the surgeon, while 15 (55.6%) cases were ruled in favor of the patient. Eighteen of the cases were the result of an appeal, of which 77.8% were decided in favor of the patient. The dates on which the actual injuries and/or surgeries occurred and the time at which the cases were settled varied dramatically. The time frame between the date of injury to the closure of the case ranged from one to 11 years, with an outlier in one case of 36 years. The mean time for case settlement was 6.6 years.

## Discussion

This study of litigation following surgery for acute traumatic facial injury found that the most common reason for proposed negligence was malocclusion after repair of a fracture or poor post-operative management. The most common defendant specialties were general surgery and plastic surgery, with the third most common being an otolaryngologist. The majority of cases and appeals were settled in favor of the patient, and each of the cases that resulted in awards of over a million dollars involved physician inattentiveness. Physicians were more likely to have a favorable verdict in the West of the US, while the most litigious state was New York. 

The ever-looming threat of litigation is on the minds of at least 63% of all physicians during their day-to-day practice, resulting in increased consultation requests and additional testing to avoid litigation [8]. Litigation may promote fear among physicians leading to overtreatment in the US healthcare system, commonly seen in the form of excessive tests or unnecessary procedures [9]. This results in potential risks including undue patient exposures, false positives on screening tests, as well as superfluous costs and societal economic burden contributing to the excessive $200 billion annual costs attributed to the US healthcare system [10]. 

A claim of malpractice relies on four main principles of medicolegal negligence: (i) establishment of professional duty between the physician and patient; (ii) breach of duty or deviation from the standard of care; (iii) injury as a direct or indirect result of the malpractice; and (iv) resultant damages [11]. Risk factors for malpractice claims are likely to be region or state-specific due to differences in governance structures, processes, and funding. Tort reform which acts to set a cap on monetary awards resulting from lawsuits may also have played a role in plaintiffs' motivations in raising a case. Of the 27 cases, only eight were decided in states that set a maximum for limiting awards. Two cases were decided in states with a hard cap on all damages (Louisiana and Nebraska), and those verdicts resulted in the lowest award compared to other cases in which damages were reported. 

There have been multiple studies in the otolaryngology literature regarding malpractice litigation, including cases involving facial paralysis, rhinoplasty, tonsillectomy, sinus surgery, and tracheotomy [12-16]. A study by Mozeika et al. similarly looked at outcomes in facial trauma malpractice from 1913 to 2016 in the Westlaw database [17]. Our study represents an updated cohort of cases, with a specific focus on the results of cases in which surgery was involved and the surgeon was party to the suit. Their paper had more emergency medicine physicians (10) and oral maxillofacial surgeons (8). They similarly cited that over 40% of the cases involved mandible fractures, however, had a higher number of panfacial fractures (36%). The case breakdown also differed in that more cases were identified in the South as well as having the highest number of settlements in favor of the defendant in the South (92%). They found a higher percentage of cases (75%) decided on behalf of the defendant. The most common reason for suit in the study was a complaint of delay or failure to diagnose, which was significantly more likely to involve emergency physicians [17]. This supports the idea that more sensitive imaging modalities should be used during the work-up of traumatic injury and may help to reduce physician liability. The current literature suggests that computed tomography (CT) imaging is superior to plain radiographs [18]. A dedicated maxillofacial CT scan with 0.6 to 1 mm cross-section imaging slices is the preferred scan and has been shown to be superior for identifying operative facial fractures, compared to head CT alone (which typically has 5-10 mm slices) [19-21].

We found that the most common reason for litigation after an acute traumatic facial injury was a functional deficit of malocclusion after operative repair of facial fractures, and a higher percentage of the claims were decided in favor of the plaintiff. The prevention of post-operative malocclusion is complex, multi-factorial, and partially operator-dependent. As with any case, a thorough physical examination and analysis of the fracture lines is important. Anatomical reduction and rigid internal fixation should be achieved in surgery and occlusion should be confirmed before closure. Early follow-up with the patient may help to detect the development of occlusal abnormalities based on patient symptoms and to determine clinical indications for revision surgery. The role of post-operative imaging in making this diagnosis has been investigated. In a study by Dewi et al., routine post-operative radiographs after open reduction internal fixation of mandibular fractures were shown to have little value in the management of patients, as the favorable/unfavorable appearance on radiographs did not correlate to symptoms, complications, or need for re-operation [22]. Durham et al. similarly reported that post-operative radiographs had a sensitivity of only 20% and a specificity of 5% to predict short-term post-operative complications for patients treated for mandible fractures [23]. Van den Bergh et al. pooled results from six studies and 1,377 patients who underwent surgery for correction of a facial fracture and found only a 0.7% surgical revision rate after the initial procedure, which was based solely on clinical findings without the use of post-operative radiographs across all studies [24]. Similarly, a prospective multicenter study by Jain et al. of 431 patients treated surgically for maxillofacial fractures found that intra-operative reduction and immediate post-operative occlusion were better indicators of reduction, fixation, and clinical outcome than immediate post-operative radiographs [25]. Therefore, the direct visualization of the fracture lines during open reduction along with confirmation of occlusion should be considered adequate evidence of proper treatment.

Physicians can potentially mitigate the risk of litigious behavior by fostering an effective doctor-patient relationship. Prior studies have shown that the rapport between physicians and their patients is a principal determinant of patients’ evaluation of their treatment [26-28]. Reasons cited for legal action have included insensitive handling and poor communication, concern for the standard of care, the need for an explanation, and holding parties accountable [26]. A study by Levinson et al. of 124 physicians found that no-claims physicians were more likely to educate their patients, spend longer in routine visits, and facilitate more patient discussion than those doctors who had legal claims brought against them [27].

Limitations of this study include its retrospective nature, small sample size, and heterogeneity of the collected data. The use of publicly available legal databases is not presumed to account for all cases and, therefore, likely underestimates the true number of litigated facial trauma cases. The reporting of summary statements and court reporting was not standardized within the databases, hence not all variables of interest could be extracted from every case.

## Conclusions

The most common reason for litigation following acute traumatic facial injuries was post-operative malocclusion after operative fixation and issues that arose during post-operative management including infection or retained packing. Ensuring intra-operative anatomical reduction at the time of surgery is the best way to prevent malocclusion. Close and diligent patient follow-up may help to recognize early post-operative complications. Fostering an effective patient-doctor relationship has been shown to reduce the incidence of litigation.
